# A single oral dose of fluralaner (Bravecto®) in dogs rapidly kills 100% of blood‐fed *Phlebotomus perniciosus*, a main visceral leishmaniasis vector, for at least 1 month after treatment

**DOI:** 10.1111/mve.12420

**Published:** 2019-11-26

**Authors:** G. Bongiorno, L. Meyer, A. Evans, N. Lekouch, R. Bianchi, C. Khoury, R. Chiummo, E. Thomas, L. Gradoni

**Affiliations:** ^1^ Unit of Vector‐borne Diseases Istituto Superiore di Sanità Rome Italy; ^2^ Clinvet SA Morocco Mohammedia Morocco; ^3^ MSD Animal Health Innovation GmbH Schwabenheim Germany

**Keywords:** Dog, fluralaner, insecticidal efficacy, *Phlebotomus perniciosus*

## Abstract

Dogs are the reservoir host of zoonotic visceral leishmaniasis (VL) caused by *Leishmania infantum* (Kinetoplastida: Trypanosomatidae). Both subclinically‐infected and sick animals can be infectious to competent phlebotomine vectors. The degree and duration of insecticidal efficacy of an oral dose of fluralaner (Bravecto®; Merck Animal Health) was determined in dogs exposed to bites of *Phlebotomus perniciosus* (Diptera: Psychodidae), a main Mediterranean vector of VL. Twelve dogs allocated to two groups of six animals each were included in a parallel‐group designed, negative‐controlled, randomized, blinded, single‐centre efficacy study. Group 2 was treated with fluralaner on day 0, and sand‐fly exposure of both groups was performed on days 1, 28 and 84. Viability of blood‐fed females was assessed up to 96 h after exposure and efficacy was measured as the survival rate of specimens fed on Group 2 versus those fed on Group 1. A mortality of 100% was recorded at 24 h in females fed on Group 2 at both days 1 and 28. Significant insecticidal efficacy was still observed on day 84, with > 50% mortality recorded by 48 h post blood meal in Group 2. Fluralaner treatment of dogs represents a promising and affordable method for reducing the pool of infected vectors in endemic settings of zoonotic VL.

## Graphical abstract

The degree and duration of insecticidal efficacy of an oral dose of fluralaner was determined in dogs exposed to bites of the *Leishmania infantum* vector *Phlebotomus perniciosus*
Sand‐fly exposure of treated and control dogs was performed up to 84 days from treatment and viability of blood‐fed insects was assessed up to 96 h after biting
A mortality of 100% was recorded in sand flies fed on treated dogs at both 1 and 28 days from treatment, and > 50% mortality was still observed on day 84

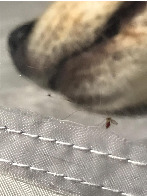

Canine leishmaniasis (CanL) is a protozoan infection/disease caused by *Leishmania infantum* (synonyms: *Leishmania chagasi*; *Leishmania infantum chagasi*) highly prevalent in all countries of the Mediterranean, Caucasian and Middle Eastern regions of the Old World, as well as in several Latin American territories (Dantas‐Torres *et al*., 2012). Infection outcomes in dogs range from subclinical to clinical, and from mild to severe disease. Although the parasite can occasionally be transmitted by non‐vectorial modes (EFSA Panel on Animal Health and Welfare, 2015), the main transmission route is by the bite of infected phlebotomine sand flies. From around 7 days after an infected blood meal, competent vectors may develop infective *Leishmania* promastigotes in the foregut, which are regurgitated into the mammal host's skin during subsequent blood meal (Bates, 2007). Both subclinical and diseased dogs can be infectious to vectors; however, the probability of disproportionately contributing to onward transmission (‘super‐spreader’ effect) increases with the duration and severity of CanL (Courtenay *et al*., 2014).

*Phlebotomus perniciosus* is the main competent vector of *L. infantum* throughout the western Mediterranean basin, including southern Europe and northwest Africa (European Centre for Disease Prevention and Control, 2019). Furthermore, *P. perniciosus* is a representative member of a larger taxonomic group, the *Larroussius* subgenus, consisting of morphologically, genetically and biologically close‐related species acting as competent vectors of *L. infantum* in central and eastern Mediterranean, such as *Phlebotomus neglectus* and *Phlebotomus tobbi* (Alten *et al*., 2016). The use of topical pyrethroids is universally considered the first‐line approach to protect healthy dogs from *Leishmania*‐infected sand‐fly bites (Maroli *et al*., 2010), whereas the management of infected/infectious dogs is a complex task because of the lack of a broad consensus in regard to the approach to take. Ideally, individual veterinary care for the management of CanL pathology should be combined with measures that aim to prevent the parasite from spreading via the vector. The latter is particularly important as part of zoonotic VL control programmes currently undertaken in several endemic countries. On the one hand, owners are reluctant to invest in pyrethroid products for dogs already infected or sick; on the other hand, antileishmanial therapies have only temporary efficacy to consistently reduce a dog's infectiousness to sand flies (Miró *et al*., 2017). Isoxazolines are a novel class of compounds targeting the central nervous system and neuromuscular junctions of arthropod vectors by blocking ligand‐gated chloride channels, thus causing the death of the arthropod (Weber & Selzer, 2016). A number of compounds have been licensed as veterinary drugs for the protection of companion animals against fleas and ticks, with long *in vivo* half‐lives that provide weeks to months of protection after a single oral administration (Kilp *et al*., 2014; Shoop *et al*., 2014). These properties attracted attention for the potential use of isoxazolines for the control of vector‐borne human diseases (Miglianico *et al*., 2018). Regarding flying insects, both the degree and duration of insecticidal efficacy were found to differ among commercially‐available isoxazoline drugs when administered in dogs, such as against *Triatoma infestans* (Loza *et al*., 2017) or the sand fly *Phlebotomus papatasi* (Gomez *et al*., 2018a), with fluralaner (Bravecto®; Merck Animal Health) shown to be the most active compound with regards to the above efficacy parameters. Recently, a clinical trial of fluralaner treatment of dogs against sand flies made use of colonized *P. papatasi* as the target species (Gomez *et al*., 2018b). However, this species may not be considered a suitable sand fly model. This is primarily because *P. papatasi* is not a competent vector of *L. infantum*, being refractory to gut development of this parasite. Moreover, earlier studies on canine topical pyrethroids (Maroli *et al*., 2010) suggest that *P. papatasi* might be less susceptible to compounds targeting the sand‐fly central nervous system than *P. perniciosus*. For example, a spot‐on formulation of 50% permethrin–10% imidacloprid killed 29% *P. papatasi* versus 49% *P. perniciosus*. These considerations prompted us to determine the onset and duration of insecticidal efficacy against *P. perniciosus* after feeding on fluralaner‐treated dogs.

A parallel‐group designed, negative‐controlled, randomized, blinded, single‐centre efficacy study was performed. Experimental dogs, consisting of male Beagles ≥ 6 months old at time of inclusion, were from a research colony in Mohammedia, Morocco; the in‐life phase study was conducted at the Clinvet facilities. Adult *P. perniciosus* specimens were from a Spanish strain maintained from June 2012 onwards at the medical entomology facilities of Istituto Superiore di Sanità, Italy, at 28 ± 1 °C and 75–80% relative humidity. The sand flies were certified for pathogen‐free status and adapted to mass‐breeding conditions with six independent colony sublines. Three‐ to 9‐day‐old specimens were pooled from the respective emerging cages and distributed at similar age proportions in cylindrical plastic pots (400 mL) fitted with a tight lid, which was perforated and the hole covered with a fine gauze holding a piece of cotton soaked with glucose saturated solution. The number of sand flies in each pot was sufficient for one dog, with the addition of a few extra specimens to compensate for deaths that occurred before the experiments. The pots were put into airtight plastic bags provided with humidified filter paper. The secondary packaging consisted of a preheated container provided with an extended temperature stability device and a digital temperature recorder, surrounded by sturdy carton box. Boxes were shipped to Morocco by air and the delivery was approximately within 3 days of shipping. Upon delivery, temperature recording was checked and the plastic bags were left unopened at 25 ± 3 °C pending the exposure of the dogs. A rough assessment of sand fly mortality in pots was performed from the outside, and a mortality rate of ≤ 10% per pot was considered acceptable for use. Ultimately, at time of the experiments, each dog was exposed to the same homogeneous population of sand flies ageing 7–14 days.

Twenty‐seven days before treatment, 14 male Beagle dogs were sedated with Domitor® (Vetoquinol, Lure, France) and their head was exposed for 1 h to bites from approximately 40 caged *P. perniciosus* females, along with five males to promote biting behaviour, aiming to assess individual attractiveness to the vector in these experimental conditions. The dogs were ranked in descending order based on live blood‐fed specimen counts; two dogs having the lowest counts were excluded from the study, and twelve dogs were included and allocated to two groups of six dogs each (Groups 1 and 2). On day 0, Group 2 dogs were treated with an oral dose of fluralaner (Bravecto® chewable tablets) at a label dose rate of 25 to 56 mg/kg body weight, whereas dogs in Group 1 remained untreated. Specific health observations were carried out at 1, 2 and 6 h after fluralaner administration to the last dog. The exposure of dogs to approximately 40–60 *P. perniciosus* females (plus 5–10 males) was performed in parallel for both groups, at similar temperature and humidity conditions in two rooms, on days 1, 28 and 84 (an intermediate assessment was initially considered for the period at around 50–60 days post treatment, although this was not possible because of technical and logistic issues). The primary criterion for efficacy determination was the survival rate of sand flies fully fed on treated dogs versus those fully fed on control dogs, with partially or marginally blood‐fed specimens not being considered. To allow optimal and durable comparisons between the two groups of sand fly specimens, females were individually recollected from exposure cages through gentle mouth aspiration, and pooled in groups of ≤ 10 specimens inside plaster‐lined plastic pots. The pots were then placed in boxes provided with saturated glucose and humidified filter paper, and maintained thereafter at 26 °C. Viability assessments were conducted at 6 h (except for day 1), and then at 24‐h intervals up to a maximum of 96 h after the end of the exposure. To assess the general viability of the sand‐fly batch employed in each experiment, the above described procedures were applied to both fed and unfed females.

The statistical significance of insecticidal efficacy results, expressed as the proportion of live fed sand‐fly counts from Group 2 versus those from Group 1, was calculated using a linear mixed model including the study group as a fixed effect and a randomization block as a random effect, with the level of significance set to α = 0.05 (two‐sided). The model used Kenward–Rogers adjustment to determine the denominator degrees of freedom.

On day 1, 78 blood‐fed specimens out of a total of 159 live females recollected from Group 2 dogs were evaluated versus 64 blood‐fed specimens/169 live females from Group 1 dogs. On day 28, blood‐fed specimens were 199/341 live females from Group 2, which were evaluated versus 146/310 from Group 1. On day 84, blood‐fed specimens were 240/326 from Group 2 and 206/339 from Group 1, respectively. Table 1 shows the mean proportions of surviving *P. perniciosus* after taking a blood meal on treated and untreated dogs. On day 1 from treatment, there was evidence of some deaths among engorged sand flies already at time of collection from Group 2 exposure cages, although none from those of Group 1. By 24 h, all blood‐fed specimens from Group 2 were dead in contrast to 11% of blood‐fed specimens from Group 1. On day 28, survival of specimens fed on Group 2 dogs was significantly lower than in those from Group 1 already at 6 h post blood meal; again, all blood‐fed specimens from Group 2 were dead at 24 h, in contrast to 3% of blood‐fed specimens from Group 1. On day 84, a number of specimens that had fed on treated dogs survived until the end of observations, with broad in‐group variations; their survival rate decreased starting from 24 h and it was significantly lower from 48 h onwards compared with specimens fed on control dogs, with a calculated insecticidal activity above 50% up to 96 h of observation.

**Table 1:** Mean ± SD proportions of surviving blood‐fed *Phlebotomus perniciosus* females and calculated insecticidal efficacy at the indicated time point.

		Mean survival proportion (%)		
Day from treatment	PBM time point evaluation (h)	Group 1	Group 2	Insecticidal efficacy (%)
1	0	100	89.6 ± 16	10.4
	24	89.2 ± 9	0.0	100
	48	76.0 ± 21		
	72	67.8 ± 21		
	96	61.1 ± 16		
28	0	96.9 ± 6	96.9 ± 6	0.0
	6	96.9 ± 6	62.6 ± 29	35.5
	24	96.9 ± 6	0.0	100
	48	93.5 ± 7		
	72	89.6 ± 8		
	96	80.6 ± 11		
84	0	100	99.4 ± 1	0.6
	6	100	91.7 ± 17	8.3
	24	100	63.6 ± 37	36.4
	48	92.7 ± 6	39.7 ± 35	57.2
	72	81.7 ± 13	37.1 ± 32	54.6
	96	66.4 ± 24	31.4 ± 29	52.7

Survival proportion per dog (%) = [(live blood‐fed females/all blood‐fed females) × 100].

Insecticidal efficacy (%) = 100 × [(mean proportion of live fed females in Group 1 − mean proportion of live fed females in Group 2)/mean proportion of live fed females in Group 1].

Group 1, six untreated dogs; Group 2, six fluralaner‐treated dogs; PBM, post blood meal.

Survival rates of unfed sand flies recollected from exposure cages were also evaluated up to 96 h after each dog's exposure. They were generally elevated and higher compared with those in blood‐fed specimens, showing some differences between each day of a dog's exposure, although not so much between experimental groups in each exposure. This may reflect batch‐to‐batch variations in mortality typically occurring between generations of colonized sand flies, and possibly different transport conditions. Blood ingestion was the only variable associated with 100% sand fly mortality in fluralaner‐treated dogs (Fig. 1).

**Figure 1:**
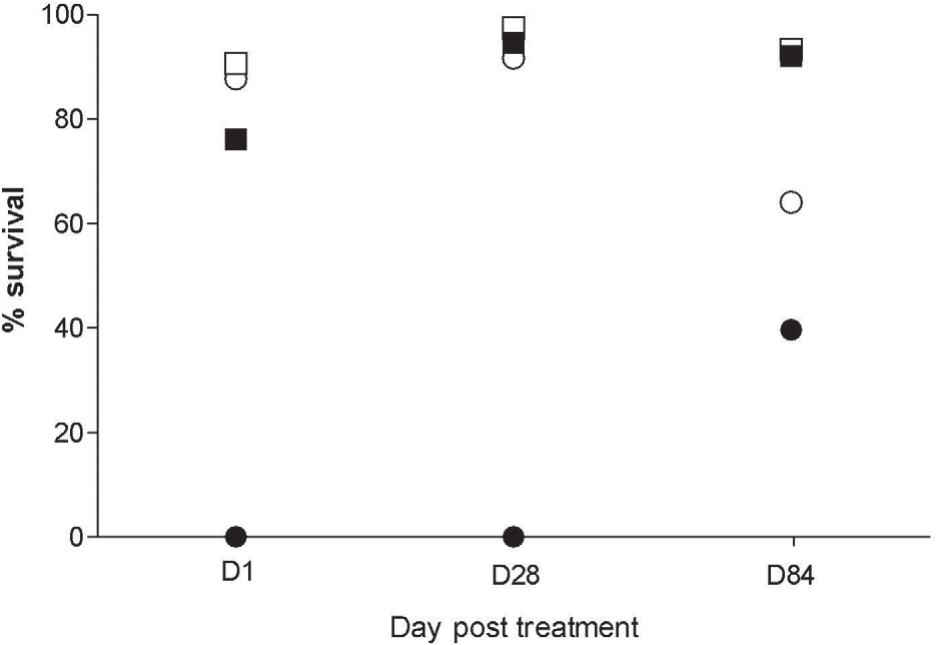
Mean proportions of surviving blood‐fed *Phlebotomus perniciosus* females 48 h post blood meal on Group 1 (untreated, 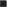) and Group 2 (treated, 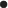) dogs, as well as of unfed females recollected from the respective exposure cages (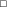; 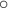) at the indicated day (D) post treatment.

The present study, although not comparing different sand fly species, provides further evidence that the susceptibility to canine insecticidal products targeting the sand‐fly central nervous system, may be higher in *P. perniciosus* than in *P. papatasi*. Using the same fluralaner formulation and doses in a study including Beagles exposed to this species, Gomez *et al*. (2018b) recorded partial survival of blood‐fed *P. papatasi* females (in the order of 6–7%) already at 17–31 days post treatment, which increased to approximately 25% at day 45.

There is > 5‐day lag period for a sand fly to be infectious after ingesting an infected blood meal. Therefore, there is a technical challenge for systemic insecticide studies in sand flies with respect to maintaining a high and durable survival of blood‐fed control specimens and detecting any delayed insecticidal effects that still have a transmission‐blocking potential. This challenge is different from most common studies with anti‐feeding treatments, where sand flies are killed by the operators immediately after a dog's exposure and scored as blood‐fed/unfed. Long‐term evaluations are needed to ensure the meaningful interpretation and statistical significance of the survival differences between treated and control groups (e.g. during descending‐phase concentrations of drugs in the canine plasma). The use of *ex vivo* canine blood from both treated and control dogs in artificial feeding systems can be prone to bacterial contamination and hence result in the rapid death of any engorged sand flies, thus allowing only a narrow temporal window to discriminate between active and non‐active drugs (Gomez *et al*., 2018a). In the present study, the survival rate of blood‐fed control specimens was very satisfactory, with a range of 61–81% at 96 h post blood meal.

The pharmacokinetics of fluralaner in canine plasma after a single oral administration showed a regular descending trend up to around 100 days post treatment, when it becomes undetectable (Kilp *et al*., 2014). It is interesting to note that a theoretical mean range of approximately 40–100 ng fluralaner/mL plasma, corresponding to 84 days post treatment, was still able to kill > 50% of *P. perniciosus* within 48 h after a blood meal. Therefore, intermediate plasma concentrations, such as those expected between 28 and 84 days from treatment, are also likely to provide elevated insecticidal activity against the vector, and therefore new studies are needed to confirm this. On the other hand, the results of the present study with respect to the three time points clearly show a coherent trend of efficacy reduction throughout the study.

In conclusion, fluralaner treatment of dogs represents a promising method for reducing the pool of infected vectors in endemic settings of zoonotic VL. Furthermore, the use of fluralaner in combination with preventive topical pyrethroid treatments, provided that this is not contraindicated for a dog's safety (Walther *et al*., 2014), could provide benefits for dogs in high‐risk areas.

## References

[mve12420-bib-0001] Alten, B. , Maia, C. , Afonso, M.O. *et al* (2016) Seasonal dynamics of phlebotomine sand fly species proven vectors of Mediterranean leishmaniasis caused by *Leishmania infantum* . PLoS Neglected Tropical Diseases, 22, e0004458. 10.1371/journal.pntd.0004458 PMC4762948 26900688

[mve12420-bib-0002] Bates, P.A. (2007) Transmission of *Leishmania* metacyclic promastigotes by phlebotomine sand flies. International Journal for Parasitology, 37, 1097–1106. 17517415 10.1016/j.ijpara.2007.04.003 PMC2675784

[mve12420-bib-0003] Courtenay, O. , Carson, C. , Calvo‐Bado, L. , Garcez, L.M. & Quinnell, R.J. (2014) Heterogeneities in *Leishmania infantum* infection: using skin parasite burdens to identify highly infectious dogs. PLoS Neglected Tropical Diseases, 8, e2583. 24416460 10.1371/journal.pntd.0002583 PMC3886905

[mve12420-bib-0004] Dantas‐Torres, F. , Solano‐Gallego, L. , Baneth, G. , Ribeiro, V.M. , de Paiva‐Cavalcanti, M. & Otranto, D. (2012) Canine leishmaniosis in the Old and New Worlds: unveiled similarities and differences. Trends in Parasitology, 28, 531–538. 22995719 10.1016/j.pt.2012.08.007

[mve12420-bib-0005] EFSA Panel on Animal Health and Welfare (2015) Scientific opinion on canine leishmaniosis. EFSA Journal, 13, 4075.

[mve12420-bib-0006] European Centre for Disease Prevention and Control (2019) Phlebotomus Perniciosus – current known distribution: January 2019. https://ecdc.europa.eu/en/publications‐data/phlebotomus‐perniciosus‐current‐known‐distribution‐january‐2019 [accessed on 8 August 2019].

[mve12420-bib-0007] Gomez, S.A. , Curdi, J.L. , Hernandez, J.A.C. *et al* (2018a) Phlebotomine mortality effect of systemic insecticides administered to dogs. Parasites & Vectors, 11, 230. 29622033 10.1186/s13071-018-2820-x PMC5887228

[mve12420-bib-0008] Gomez, S.A. , Lucientes, J. , Castillo, J.A. *et al* (2018b) A randomized, blinded, controlled trial to assess sand fly mortality of fluralaner administered orally in dogs. Parasites & Vectors, 11, 627. 30518412 10.1186/s13071-018-3231-8 PMC6282346

[mve12420-bib-0009] Kilp, S. , Ramirez, D. , Allan, M.J. , Roepke, R.K. & Nuernberger, M.C. (2014) Pharmacokinetics of fluralaner in dogs following a single oral or intravenous administration. Parasites & Vectors, 7, 85. 24606874 10.1186/1756-3305-7-85 PMC3975451

[mve12420-bib-0010] Loza, A. , Talaga, A. , Herbas, G. *et al* (2017) Systemic insecticide treatment of the canine reservoir of *Trypanosoma cruzi* induces high levels of lethality in *Triatoma infestans*, a principal vector of Chagas disease. Parasites & Vectors, 10, 344. 28724448 10.1186/s13071-017-2278-2 PMC5518140

[mve12420-bib-0011] Maroli, M. , Gradoni, L. , Oliva, G. *et al* (2010) Guidelines for prevention of leishmaniasis in dogs. Journal of the American Veterinary Medical Association, 236, 1200–1206. 20513198 10.2460/javma.236.11.1200

[mve12420-bib-0012] Miglianico, M. , Eldering, M. , Slater, H. *et al* (2018) Repurposing isoxazoline veterinary drugs for control of vector‐borne human diseases. Proceedings of the National Academy of Sciences of the United States of America, 115, E6920–E6926. 29967151 10.1073/pnas.1801338115 PMC6055183

[mve12420-bib-0013] Miró, G. , Petersen, C. , Cardoso, L. *et al* (2017) Novel areas for prevention and control of canine leishmaniosis. Trends in Parasitology, 33, 718–730. 28601528 10.1016/j.pt.2017.05.005

[mve12420-bib-0014] Shoop, W.L. , Hartline, E.J. , Gould, B.R. *et al* (2014) Discovery and mode of action of afoxolaner, a new isoxazoline parasiticide for dogs. Veterinary Parasitology, 201, 179–189. 24631502 10.1016/j.vetpar.2014.02.020

[mve12420-bib-0015] Walther, F.M. , Fisara, P. , Allan, M.J. , Roepke, R.K. & Nuernberger, M.C. (2014) Safety of the concurrent treatment of dogs with Bravecto™ (fluralaner) and Scalibor™ protectorband (deltamethrin). Parasites & Vectors, 7, 105. 24646450 10.1186/1756-3305-7-105 PMC3972964

[mve12420-bib-0016] Weber, T. & Selzer, P.M. (2016) Isoxazolines: a novel chemotype highly effective on ectoparasites. ChemMedChem, 11, 270–276. 26733048 10.1002/cmdc.201500516

